# Population Differentiation and Hybridisation of Australian Snubfin (*Orcaella heinsohni*) and Indo-Pacific Humpback (*Sousa chinensis*) Dolphins in North-Western Australia

**DOI:** 10.1371/journal.pone.0101427

**Published:** 2014-07-02

**Authors:** Alexander M. Brown, Anna M. Kopps, Simon J. Allen, Lars Bejder, Bethan Littleford-Colquhoun, Guido J. Parra, Daniele Cagnazzi, Deborah Thiele, Carol Palmer, Celine H. Frère

**Affiliations:** 1 Murdoch University Cetacean Research Unit, School of Veterinary and Life Sciences, Murdoch University, Perth, Western Australia, Australia; 2 Centre for Ecology and Conservation, University of Exeter, Penryn, Cornwall, United Kingdom; 3 Marine Evolution and Conservation, Centre for Ecological and Evolutionary Studies, University of Groningen, Groningen, The Netherlands; 4 Cetacean Ecology, Behaviour and Evolution Lab, School of Biological Sciences, Flinders University, Adelaide, South Australia, Australia; 5 South Australian Research and Development Institute, Adelaide, South Australia, Australia; 6 Marine Ecology Research Centre, School of Environment, Science and Engineering, Southern Cross University, Lismore, New South Wales, Australia; 7 Fenner School of Environment & Society, Australian National University, Canberra, Australian Capital Territory, Australia; 8 Marine Ecosystems, Flora and Fauna Division, Department of Land Resource Management, Palmerston, Northern Territory, Australia; 9 Research Institute for the Environment and Livelihoods, Charles Darwin University, Darwin, Northern Territory, Australia; 10 GeneCology Research Centre, University of the Sunshine Coast, Maroochydore DC, Queensland, Australia; Natural History Museum of Denmark, Denmark

## Abstract

Little is known about the Australian snubfin (*Orcaella heinsohni*) and Indo-Pacific humpback (*Sousa chinensis*) dolphins (‘snubfin’ and ‘humpback dolphins’, hereafter) of north-western Australia. While both species are listed as ‘near threatened’ by the IUCN, data deficiencies are impeding rigorous assessment of their conservation status across Australia. Understanding the genetic structure of populations, including levels of gene flow among populations, is important for the assessment of conservation status and the effective management of a species. Using nuclear and mitochondrial DNA markers, we assessed population genetic diversity and differentiation between snubfin dolphins from Cygnet (n = 32) and Roebuck Bays (n = 25), and humpback dolphins from the Dampier Archipelago (n = 19) and the North West Cape (n = 18). All sampling locations were separated by geographic distances >200 km. For each species, we found significant genetic differentiation between sampling locations based on 12 (for snubfin dolphins) and 13 (for humpback dolphins) microsatellite loci (*F*_ST_ = 0.05–0.09; *P*<0.001) and a 422 bp sequence of the mitochondrial control region (*F*_ST_ = 0.50–0.70; *P*<0.001). The estimated proportion of migrants in a population ranged from 0.01 (95% CI 0.00–0.06) to 0.13 (0.03–0.24). These are the first estimates of genetic diversity and differentiation for snubfin and humpback dolphins in Western Australia, providing valuable information towards the assessment of their conservation status in this rapidly developing region. Our results suggest that north-western Australian snubfin and humpback dolphins may exist as metapopulations of small, largely isolated population fragments, and should be managed accordingly. Management plans should seek to maintain effective population size and gene flow. Additionally, while interactions of a socio-sexual nature between these two species have been observed previously, here we provide strong evidence for the first documented case of hybridisation between a female snubfin dolphin and a male humpback dolphin.

## Introduction

Maintaining genetic diversity is a key objective of biodiversity conservation [1]. Species of conservation concern are often characterised by small, fragmented populations with restricted gene flow and low genetic diversity [2], [3]. Small and fragmented populations with severely restricted gene flow are more vulnerable to the accumulation of deleterious mutations, the loss of genetic diversity through random genetic drift, and inbreeding depression than single populations of the same effective population size [4]–[7]. Additionally, further isolation and decline of fragmented populations within species may, through mate limitation, increase the probability of hybridisation with related, sympatric species (e.g. [8], [9]). These processes may reduce the fitness of populations and impede their ability to adapt to environmental change, resulting in a reduced evolutionary potential and greater risk of extinction [10]–[12]. Understanding the genetic structure of populations, including levels of gene flow among populations and genetic diversity, is therefore important for the assessment of a species’ conservation status as well as the effective management of a species, particularly where anthropogenic activities may contribute to population fragmentation [13].

Inshore dolphins occupying coastal and estuarine areas frequently overlap with areas of high human activity, exposing them to a variety of threats, including habitat loss and degradation, acoustic disturbance, vessel strikes, pollution and incidental capture in fisheries [14]. These threats, combined with the late maturation, slow reproduction, often low abundance and restricted ranges of inshore dolphins, have resulted in priority conservation status being afforded to a number of geographically isolated populations [15]–[17].

The Australian snubfin dolphin (*Orcaella heinsohni*, ‘snubfin dolphin’ hereafter) occurs throughout tropical coastal waters of northern Australia and, potentially, Papua New Guinea [18]. The Indo-Pacific humpback dolphin (*Sousa chinensis*, ‘humpback dolphin’ hereafter) occurs in tropical and temperate inshore waters throughout the Indian and western Pacific Oceans [19], although genetic and morphological data strongly suggest that those in Australian waters are distinct from those in Southeast Asia [20], [21]. Throughout their ranges, the conservation status of both species was assessed as ‘near threatened’ by the IUCN, with caveats noting that additional data would likely result in an elevated status [22], [23].

Despite their ‘near threatened’ conservation listing, the distribution, abundance and population structure of snubfin and humpback dolphins are poorly understood throughout the majority of their ranges in Australian waters. This lack of information is impeding rigorous assessment of their conservation status [24]–[26]. Studies to date have been largely restricted to the east coast of Australia, primarily in waters adjacent to population centres in Queensland, where snubfin and humpback dolphins exhibit a discontinuous contemporary distribution of small populations of 50–100 animals [24], [26]–[30]. These populations have relatively small ranges of approximately 200–350 km^2^ and a preference for inshore habitats of waters <15 m deep and within 5 km of the coast [26], [28]–[32].

Snubfin and humpback dolphins are sympatric throughout most of their distribution in Australia [28], [33], which also overlaps that of Indo-Pacific bottlenose dolphins (*Tursiops aduncus*, ‘bottlenose dolphin’ hereafter). Where species are sympatric, inter-species associations and inter-species mating may facilitate hybridisation. This phenomenon has been reported between several cetacean species (review in [34], [35]), particularly among small cetaceans [9], [36]–[38]. To date, no hybrids have been confirmed between snubfin, humpback, or bottlenose dolphins. However, associations between snubfin and humpback dolphins have been reported at several locations along the Queensland coast [30], [39], as have associations between humpback and bottlenose dolphins, and snubfin and bottlenose dolphins in north-western Australia [40], [41]. In Cleveland Bay, Queensland, the majority (58%, n = 11) of snubfin-humpback dolphin associations were of an aggressive-sexual nature where, in all cases, humpback dolphins were identified as the aggressors [39]. Although the benefits and costs of these interactions are not fully understood, they suggest that inter-specific mating is possible.

Wild hybridisation is typically a conservation concern; when mediated by anthropogenic translocation of species and habitat modification, it has led to the extinction of many animal species and is particularly problematic for species of low abundance [42], [43]. Several studies have reported hybridisation events among mammalian species within modified habitats and/or where populations have undergone a decline (e.g. [8], [44], [45]). However, there is evidence that natural hybridisation may play an important role in the evolution of animals (e.g. [46], [47]), as has long been recognised for plants [48].

Examining the structure of populations in the marine environment presents a particular challenge due to the absence of obvious barriers to gene flow, and the highly mobile nature of many marine species. Robust demographic and movement data are often costly and logistically difficult to acquire, while similar challenges exist for the identification of hybridisation through morphological data and observations of species interactions. To this end, molecular tools have been employed to address a variety of questions in mobile marine taxa of conservation and management importance, such as teleost fish (e.g. [49], [50]), elasmobranchs (e.g. [51]), marine reptiles (e.g. [52], [53]) and marine mammals (e.g. [54]–[56]). In marine mammals, analyses of molecular markers have often contributed towards the identification of appropriate management units to inform decision-makers (e.g. [57]–[59]), including the identification of cryptic taxa and genetically-isolated populations of conservation concern (e.g. [18], [60]–[62]). Furthermore, molecular tools have permitted the investigation of hybridisation in the absence of other conclusive evidence (e.g. [9], [38]).

Molecular studies of snubfin and humpback dolphins in Australia are largely restricted to investigations of taxonomy [18], [20], [21], [63], [64]. The exception is Cagnazzi [30], who examined genetic population structure based on microsatellites of both species sampled at several locations along the Queensland coast. For snubfin dolphins, no structure was found between three populations within a 200 km stretch of coast, but significant differentiation was found between this region and a population approximately 600 km distant. The latter population, which numbers fewer than 100 individuals and is threatened by loss of habitat from port development, has been suggested as qualifying for ‘endangered’ status under IUCN Red List criteria for regional populations [26]. For humpback dolphins, significant genetic differentiation was detected between almost all putative populations, even when separated by only a few kilometres, such as in the Great Sandy Strait [30]. In contrast, a recent study in Chinese waters found no evidence of genetic population structure in humpback dolphins among three resident populations along a *ca.* 1,000 km stretch of coastline [65].

The lack of information on the genetic population structure of snubfin and humpback dolphins is of particular concern in the north-west of Australia, where data deficiencies are coupled with a resources extraction boom, resulting in widespread and large-scale habitat modification of the inshore environment associated with port development [25], [40]. The development of the coastal zone may introduce anthropogenic barriers to dispersal and cause fragmentation of inshore dolphin populations. However, in the absence of any understanding of the genetic diversity or connectivity between populations, the likelihood or significance of these potential effects on inshore dolphins remains unknown. Information on the genetic population structure of these species in this region is essential to determining an appropriate management scale at which to assess potential anthropogenic effects and inform conservation strategies.

In this study, we used mitochondrial DNA (mtDNA) sequence data and nuclear microsatellite markers to examine the genetic diversity and structure of snubfin and humpback dolphins among a limited number of study sites in north-western Australia. In addition to population structure, we also investigated the possible existence of hybrid dolphins across the study area.

## Materials and Methods

### Ethics statement

Field data collection took place under permits from the WA Department of Local Government Research and Development (U6/2010–2011), the Department of Agriculture and Food (U6/2012–2014), Department of Environment and Conservation (now Department of Parks and Wildlife) WA (SF007596, SF008480, SF008825, SF009119), WA Police (9990071), and with approval from Murdoch, Flinders and the Australian National University Animal Ethics Committees (W2342/10, E297 and A2011/50).

### Study sites and sample collection

**Figure 1:**
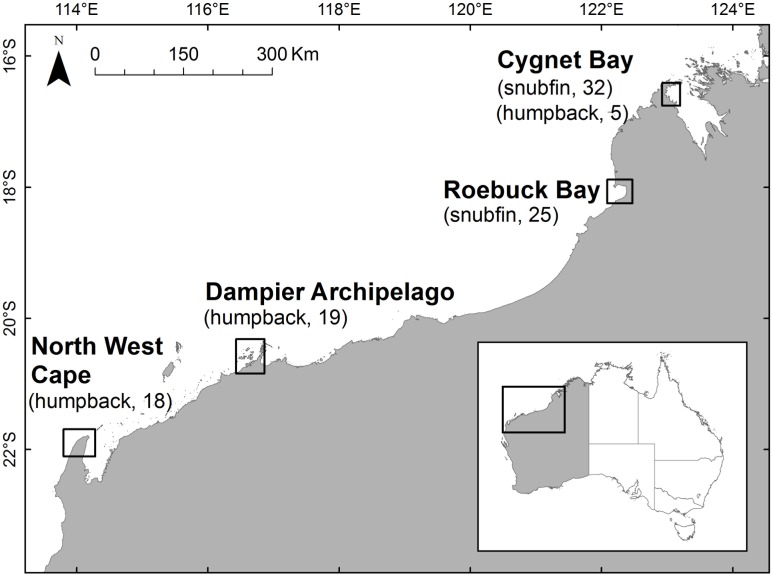
Biopsy sampling locations and sample sizes of Australian snubfin and Indo-Pacific humpback dolphins in north-western Australia.

A total of 110 skin tissue samples were obtained from free-ranging dolphins across north-western Australia between 2008 and 2013 using a biopsy darting system from small research vessels [66]. Snubfin dolphin samples were obtained from Cygnet Bay and Roebuck Bay, and humpback dolphin samples were obtained from Cygnet Bay, the Dampier Archipelago and the North West Cape (Figure 1). To assist in identifying the parental species of a suspected hybrid, we also collected biopsy samples from Indo-Pacific bottlenose dolphins from Cygnet Bay, so as to include all three dolphin species regularly encountered in Cygnet Bay into our analyses. Tissue samples were stored in either 100% ethanol or saturated NaCl/20% dimethyl sulfoxide [67]. Sampled sites represent those accessible by small research vessel and where snubfin or humpback dolphins were sufficiently approachable to distances suitable for successfully obtaining biopsy samples. Samples were primarily collected on an opportunistic basis during research on bottlenose dolphin (*Tursiops* spp.) population structure across north-western Australia [40], and also in parallel to demographic studies of snubfin and humpback dolphins at these locations (Brown *et al*., unpublished data; Thiele *et al*., unpublished data).

### Genetic analyses

Genomic DNA was extracted using the DNeasy Blood & Tissue Kit (Qiagen) following the manufacturer’s instructions. Sex was determined genetically using sex chromosome-specific primers. Loci ZFX and SRY [68] were coamplified in a single PCR reaction. PCR products were run on a 1.5% agarose gel and sex determined based on the number of different fragments amplified.

Mitochondrial DNA (mtDNA) haplotypes were assigned based on a 422 base pair (bp) sequence. The fragment was amplified by the primers dlp1.5 and dlp5 [69]. We followed the PCR conditions described in Bacher *et al.* [70]. Haplotypes were assigned with the software Geneious R6.1 (Biomatters).

We amplified 14 microsatellite loci in four 10 µl volume multiplex PCRs using Qiagen Multiplex Kit™ (Qiagen). The microsatellite markers used here were: DIrFCB1, DIrFCB4 [71], LobsDi_7.1, LobsDi_9, LobsDi_19, LobsDi_21, LobsDi_24, LobsDi_39 [72], SCA9, SCA22, SCA27 SCA39 [73], TexVet5, TexVet7 [74]. We followed the PCR conditions as described in Frère *et al.* [75]. The single stranded PCR products were run on an ABI 3730 DNA Sequencer (Applied Biosystems). Alleles were scored with Genemapper Software 3.7 (Applied Biosystems). We identified duplicate samples, i.e. samples that were genotyped for at least 10 microsatellite loci and matched 95%, using the Microsatellite Toolkit [76] and, from these, we retained the sample with the most complete genotype. Microsatellites were checked for Hardy-Weinberg equilibrium and linkage disequilibrium in GenePop [77].

Several measures of population differentiation were calculated for the sampled study sites. The suspected hybrid and the bottlenose dolphins were excluded from all analyses comparing population structure and diversity within snubfin and humpback dolphins. We calculated F_ST_ values (for microsatellites and mtDNA) and Φ_ST_ values (for mtDNA) in Arlequin [78].

Contemporary migration rates were calculated in BayesAss 1.3 [79] using 10^7^ iterations, a burn-in length of 10^6^ and a sampling interval of 1,000 steps. We performed three runs per species with different seeds to confirm that similar mean posterior migration rates and 95% confidence intervals were obtained. An admixture model without information on sampling location was run in STRUCTURE (version 2.2.3 [80], [81]) to examine differentiation patterns between populations, with a burn-in length of 10^5^ and 10^6^ Markov chain Monte Carlo (MCMC) steps. The most likely number of genetically homogeneous clusters (if greater than two) was determined based on 10 iterations for each population (k) = 1–4 by calculating Δk, an ad hoc statistic proposed by Evanno *et al.* [82]. Δk was calculated in STRUCTURE HARVESTER (Web v0.6.93 [83]). We also compared STRUCTURE results to those of the recently published software FLOCK (FLOCK_MSAT 3.0 [84]) using default parameters. Compared to the MCMC-based STRUCTURE, FLOCK uses an iterative method which makes it faster and computationally more efficient.

We calculated the effective population (*N_e_*) sizes for snubfin dolphins based on the linkage disequilibrium method using LNDe v1.31 [85]. For small effective population sizes of <500, the linkage disequilibrium (LD) method has shown to be reliable with the use of 10–20 microsatellite loci and samples of 25–50 individuals [86]. We did not calculate *N_e_* for humpback dolphins because the number of samples per population was less than 25.

An underlying assumption of the linkage-disequilibrium method of estimating *N_e_* is non-overlapping generations. This assumption is violated within the long-lived, polygamous populations examined here, and may lead to a downward bias in estimates of *N_e_* [86]–[88]. Despite this, Robinson & Moyer [87] showed that for populations with small *N_e_*, the linkage-disequilibrium method performed relatively well for species with overlapping generations under a variety of life history scenarios and sampling strategies. Random sampling of mature individuals, as was the case in the current study, has been shown to produce the best estimates of *N_e_* by LD [87]. The lowest allele frequency considered in the analyses was set to 0.03 to ensure that single copy alleles were filtered out; *N_e_* estimates were correspondingly corrected for downward bias by multiplying the estimate by 1.25 [86], [89]. Due to the paucity of information of snubfin dolphin life history traits, we used a correction factor suggested for bottlenose dolphins [89].

We also tested whether any population has recently undergone a bottleneck using a graphical method to detect allele frequency distortion [90] and the software BOTTLENECK (v1.2.02 [91]). We specified 1,000 iterations and used Wilcoxon sign rank tests to assess significance. BOTTLENECK v1.2.02 provides results for three models of the generation of new alleles; the stepwise mutation model (SMM), the infinite allele model (IAM) and the two-phased model of mutation (TPM). In the software manual, the authors recommend the use of TPM for microsatellite datasets; in their paper [92], by contrast, IAM is recommended for microsatellites with fewer than 3 bp repeats. However, TPM is not discussed in the paper.

### Hybrid investigation

In Cygnet Bay, we encountered a dolphin that, phenotypically, could not be identified as a humpback, snubfin or bottlenose dolphin. All three of these species are regularly encountered within Cygnet Bay. To confirm hybrid status and to identify the suspected hybrid’s parental species, we compared the suspected hybrid’s mtDNA haplotype to those of humpback, snubfin and bottlenose dolphins. We also compared the microsatellite genotype of the suspected hybrid to alleles found in the three dolphin species at Cygnet Bay. By doing so, we could assign the parental species of the suspected hybrid based on species-specific alleles. Furthermore, we ran STRUCTURE to obtain a measure of likelihood to which species the suspected hybrid belongs. All samples collected at Cygnet Bay were included in the STRUCTURE analysis using the same parameters as above.

Microsatellite genotypes used in this study are available in the supplementary material and mtDNA haplotype sequences have been archived on GenBank (Accession numbers KJ530719–KJ530740).

## Results

### Population differentiation

After having removed ten duplicate samples from across the dataset, we conducted the analyses with the following populations and sample sizes: snubfin dolphins from Cygnet Bay (n = 32) and Roebuck Bay (n = 25), and humpback dolphins from Cygnet Bay (n = 5), the Dampier Archipelago (n = 19) and the North West Cape (n = 18). We do not present F_ST,_ Φ_ST_ or contemporary migration rate values for humpback dolphins from Cygnet Bay due to the low sample size. Additionally, we collected one sample of a suspected hybrid and six samples from bottlenose dolphins from Cygnet Bay.

**Figure 2:**
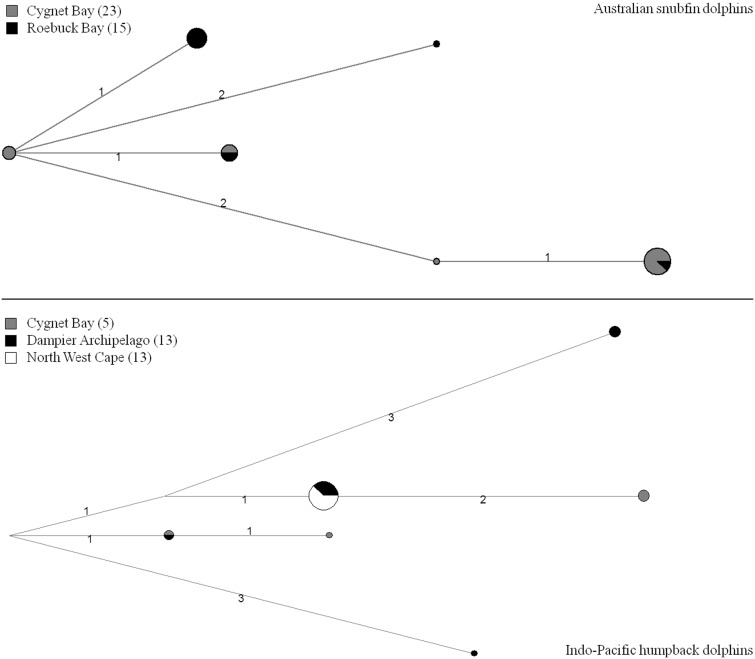
mtDNA networks for snubfin and humpback dolphins. Sample sizes are shown in parentheses. Branch numbers indicate the number of nucleotide differences between mtDNA haplotypes.

**Figure 3:**
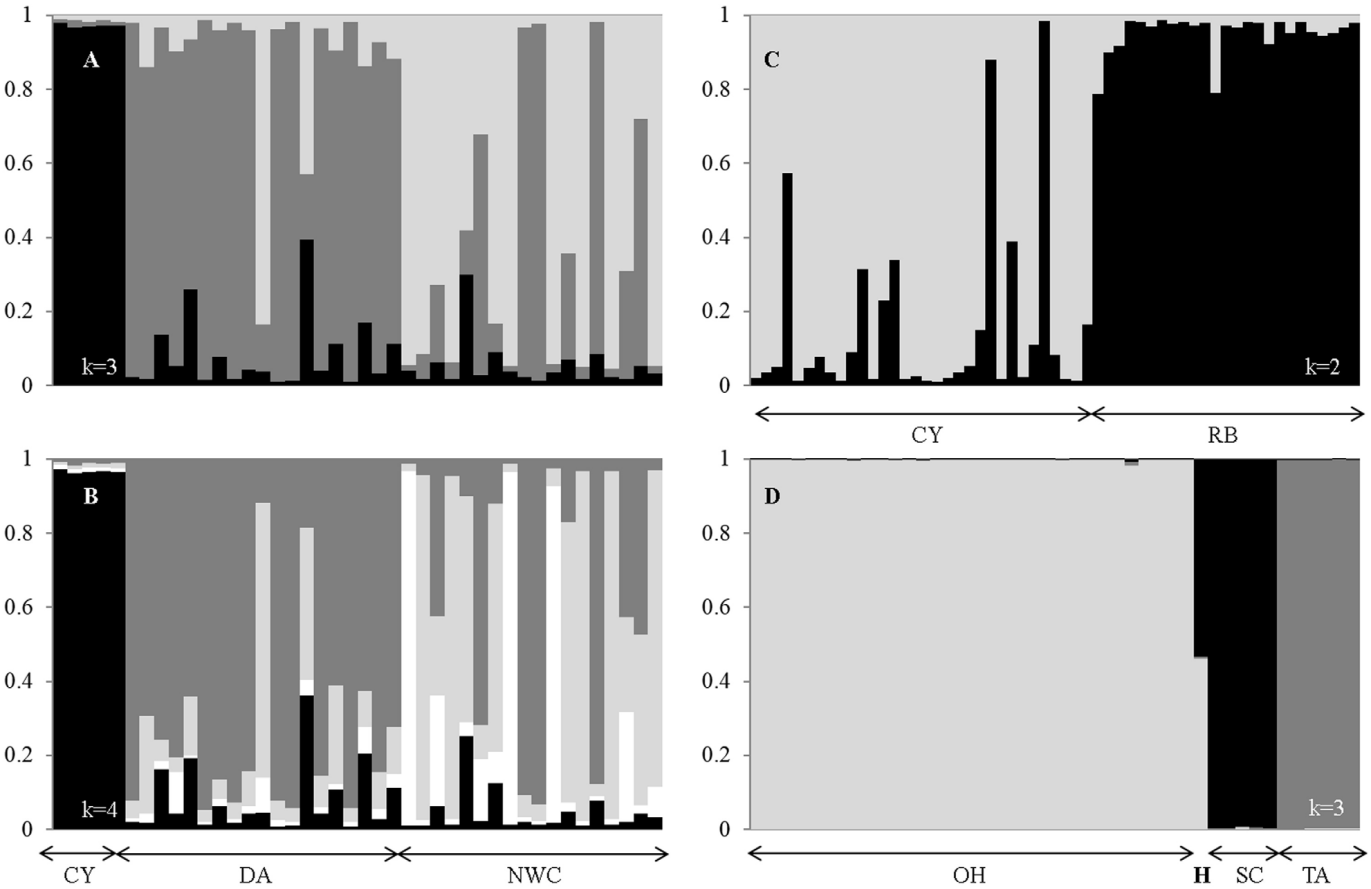
Structure plots for humpback dolphins where k = 3 (A) and k = 4 (B), for snubfin dolphins (C), and the three regularly encountered dolphin species at Cygnet Bay (D). k = number of clusters. Each bar on the x-axis corresponds to an individual. The y-axis indicates the proportion of population/species membership. OH = snubfin dolphins, SC = humpback dolphins, CY = Cygnet Bay, DA = Dampier Archipelago, NWC = North West Cape, RB = Roebuck Bay, H = suspected hybrid, TA = bottlenose dolphin.

Twelve of the 14 genotyped microsatellite loci were polymorphic in snubfin dolphins and 13 microsatellite loci were polymorphic in humpback dolphins (Table 1, Table S4). On average we genotyped 95% of loci per individual. For both species, none of the microsatellite loci appeared out of Hardy-Weinberg Equilibrium after sequential Bonferroni correction [93], nor linked after sequential Bonferroni correction. We found six mtDNA haplotypes each in snubfin and humpback dolphins (Figure 2). Within species, all population pairs were significantly differentiated based on microsatellites (F_ST_ = 0.05–0.09) and mtDNA loci (F_ST_ = 0.50–0.70, Φ_ST_ = 0.17–0.45) (Table 2). STRUCTURE assigned most individuals sampled at the same location to the same cluster (Figure 3A–3C). For snubfin dolphins, Δk analysis and FLOCK showed that the most likely k was ≤2 (Figure S1). For humpback dolphins, the most likely number of k was four based on STRUCTURE (Figure S1) and three based on FLOCK. Three equals the number of sampled populations.

**Table 1:** Microsatellite characteristics for snubfin and humpback dolphins.

	N_A_	N_E_	F_IS_	H_E_	H_O_
**Snubfin dolphins**					
Cygnet Bay	4.25	2.65	0.00	0.57	0.58
Roebuck Bay	4.25	2.88	−0.01	0.58	0.60
**Humpback dolphins**					
Dampier Archipelago	3.73	2.09	−0.07	0.44	0.46
North West Cape	3.58	2.16	0.06	0.40	0.35

N_A_ = Number of Alleles, N_E_ = Number of effective Alleles, F_IS_ = Inbreeding Coefficient, H_E_ = expected heterozygosity, H_O_ = observed heterozygosity.

Numbers are averages over polymorphic loci. See Tables A1 and A2 for locus specific microsatellite characteristics.

**Table 2:** Genetic differentiation of mtDNA and microsatellite loci.

	Measure of differentiation	mtDNA	microsatellites
**Snubfin dolphins**	***F*** **_ST_**	0.500**	0.091**
**(CY-RB)**	**Φ_ST_**	0.446**	na
**Humpback dolphins**	***F*** **_ST_**	0.699**	0.046**
**(DA-NWC)**	**Φ_ST_**	0.167*	na

Asterisks indicate *P* values (*P<0.05, **P<0.001). CY = Cygnet Bay, RB = Roebuck Bay, DA = Dampier Archipelago, NWC = North West Cape. For the mtDNA based estimates a lower sample size was used for both species; 15 samples from RB, 23 samples from CY, and 13 samples each from DA and NWC.

Contemporary migration rates (i.e. within the last few generations) revealed an estimated proportion of 0.04 (95% CI 0.01–0.10) of snubfin dolphins in Cygnet Bay derived from Roebuck Bay and 0.03 (0.00–0.08) of Roebuck Bay individuals derived from Cygnet Bay. For humpback dolphins, we estimated a proportion of 0.01 (0.00–0.06) individuals from the Dampier Archipelago derived from the North West Cape and 0.13 (0.03–0.24) of North West Cape individuals derived from the Dampier Archipelago.

### Effective population size and evidence of bottlenecks

For snubfin dolphins, *N_e_* (95% CI) was estimated to be 49.1 (28.6–112.1) for Cygnet Bay and 56.0 (24.3–77180.6) for Roebuck Bay. The wide confidence intervals are revisited in the discussion. We obtained conflicting results on recent bottlenecks depending on the method used (see Table S3 for *P* values and Figure S3 for visualisations of potential mode shifts).

### Suspected hybrid

**Figure 4:**
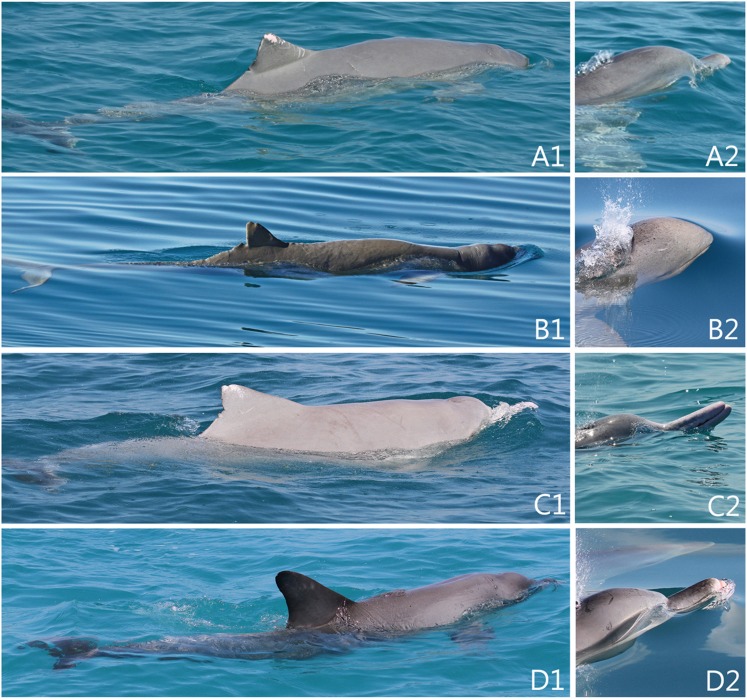
Images of hybrid (A1–2), adult snubfin (B1–2), humpback (D1–2) and bottlenose (D1–2) dolphins encountered at Cygnet Bay. Left images show relative dorsal proportions; right images compare head/rostrum characteristics.

The Cygnet Bay individual that could not be visually assigned to species level exhibited a length, girth and light grey colouration typical of adult humpback dolphins in the region. The low, triangular dorsal fin was also indicative of a humpback dolphin, although the position of the dorsal fin was posterior to the mid-point of the body, as in a snubfin dolphin. The surfacing movement was comparable to that of a snubfin dolphin, tilting back the head to breathe, with faint neck creases visible (although without the prominent sunken post-cranial region of a snubfin dolphin). A short rostrum was visible, being noticeably shorter than that of a bottlenose dolphin and far shorter than that of a humpback dolphin (Figure 4).

Over four×one month seasons of photo-identification and biopsy sampling surveys at Cygnet Bay from 2012–2013, the suspected hybrid was observed 22 times on 17 different days (Brown *et al.*, unpublished data). Over these observations, a total of eight hours were spent in the presence of the suspected hybrid; 23% of the time the animal was alone (defined as>100 m from any other individual), 77% in close (<10 m) association with one or more snubfin dolphins. Only two brief close associations with humpback and bottlenose dolphins were recorded; in both encounters the suspected hybrid was also in close association with one or more snubfin dolphins. The majority of associations with snubfin dolphins were small groups (<5 individuals) with female individuals (confirmed through genetics or presence of dependent calf). In September 2013, the suspected hybrid was observed on four occasions in larger snubfin dolphin groups (>10 individuals) of mixed sex.

Genetic analyses revealed that the individual was a female and supported its status as a hybrid. The comparison of the hybrid’s genotype to alleles found in the three resident dolphin species within Cygnet Bay indicated the majority of alleles (84.4%) found were species-specific. The hybrid shares at least one allele of each microsatellite locus with snubfin dolphins and at least one allele of each microsatellite locus for 11 out of the 14 loci with humpback dolphins (Table 3). At one locus, the hybrid is homozygote and this allele is only shared with snubfin dolphins. At five loci, the hybrid shares an allele with bottlenose dolphins, however, only one of them has not been found in either snubfin or humpback dolphins (Table 3).

**Table 3:** Alleles shared by the suspected hybrid and the three resident dolphin species at Cygnet Bay.

	Allele 1			Allele 2			hybrid homozygote	Support of hybrid hypothesis? If not, what are potential explanations?
Locus	OH	SC	TA	OH	SC	TA		
**DIrFCB4**	y				y			y
**DIrFCB5**	y				y			y
**Lobs7.1**	y				y			y
**LobsDi9**	y			y			y	n; null allele or allele might not have been sampled in SC due to small sample size or rare allele
**LobsDi19**	y				y			y
**LobsDi21**	y		y		y			y
**LobsDi24**	y			y				n; allele 1 or allele 2 might not have been sampled in SC due to small sample size or rare allele
**LobsDi39**	y				y	y		y
**SCA9**	y				y			y
**SCA22**	y	y	y			y		n; allele 2 might not have been sampled in SC and/or OH due to small sample size, or rare allele
**SCA27**	y		y		y			y
**SCA39**	y		y		y	y		y
**Tex5**	y	y		y	y		y	y
**Tex7**	y				y			y

OH = snubfin dolphin, SC = humpback dolphin, TA = bottlenose dolphin, y = yes, n = no.

STRUCTURE analyses including snubfin, humpback and bottlenose dolphins from Cygnet Bay estimated that the sample originated to 53.4±0.05% (mean of 10 iterations ± SD, k = 3) from a snubfin dolphin, to 46.2±0.05% from a humpback dolphin and to 0.4±0.00% from a bottlenose dolphin (as indicated by the proportion of shading in individual bars in Figure 3D). The mtDNA haplotype of the suspected hybrid matched a haplotype found in snubfin dolphins (Figure S4), suggesting that she most likely had a snubfin dolphin mother. The STRUCTURE results and allele comparisons suggest a humpback dolphin father.

From all samples included in this study, other than the hybrid, only one other, a male snubfin dolphin from Roebuck Bay, showed some signs of mixed species ancestry. Images of this individual suggest a normal snubfin dolphin phenotype. STRUCTURE assigned this individual by 16.7% (10 iterations, SD = 0.00) to humpback dolphin (Figure S2) and 83.1% (0.00) to snubfin dolphin. This is suggestive of post-F1 hybrid status, although the small number of microsatellite markers used in this study restricts our interpretation of such results.

## Discussion

### Population differentiation

We found that snubfin and humpback dolphins showed significant levels of population structure at both the mitochondrial and microsatellite DNA level between the sampling locations. Significant F_ST_ and Φ_ST_ values for snubfin dolphins between Roebuck Bay and Cygnet Bay provide genetic evidence for the presence of discrete populations with limited gene flow. The two populations shared two out of six mtDNA haplotypes and 15 private microsatellite alleles were detected (Table S1). Within each of these sampling locations, STRUCTURE assigned most snubfin individuals to the same cluster. However, three individuals (9%) at Cygnet Bay were predominately assigned to the Roebuck Bay cluster, suggesting that they were Roebuck Bay migrants or of migrant ancestry (Figure 3).

Humpback dolphins from the Dampier Archipelago and the North West Cape also exhibit significant population structure with limited gene flow. Significant F_ST_ and Φ_ST_ values were obtained between two sampling locations, and the results of STRUCTURE and FLOCK assigned the majority of animals at these two locations to separate clusters. However, there was some evidence of movement of individuals between sites, particularly from the Dampier Archipelago to the North West Cape, the latter of which included five individuals (26%) predominately assigned to the dominant cluster at the Dampier Archipelago (Figure 3). Humpback dolphins occur along a further 400+km of coastline south of the North West Cape [40]. The results of STRUCTURE at k = 4 further illustrate admixture within North West Cape humpback dolphins and suggest the existence of a potential fourth, not yet sampled, humpback population, possibly to the south of the North West Cape. The sample size for humpback dolphins from Cygnet Bay (n = 5) was too small to calculate meaningful F_ST_ and Φ_ST_ values with samples from the other two locations. However, Cygnet Bay humpback dolphins seem to be genetically differentiated from the other two sampling locations, based on the strong partitioning in the STRUCTURE results. Based on all three sampling sites, two out of six mtDNA haplotypes were shared among dolphins from two out of three different sampling locations, and there were 16 private microsatellite alleles detected (Table S2).

For both species, most contemporary migration rates were low, with estimated proportions of migrants ≤0.04 between sites. Confidence intervals around these estimates were wide, owing to the relatively small sample sizes. However, for most sites, the upper confidence interval of the proportion of migrants was ≤0.1. The exception was migration rates of humpback dolphins from the Dampier Archipelago to the North West Cape, which were slightly higher at 0.13 (95% CI 0.03–0.24) – a result supported by the greater admixture of humpback dolphins at the North West Cape revealed by STRUCTURE. Our confidence in this apparent directionality of gene flow for humpback dolphins between the Dampier Archipelago and the North West Cape is limited by largely overlapping confidence intervals between the two estimates of migration rates. A greater number of samples is required to further investigate the potential source-sink pattern of population structure (e.g. [94]).

Although limited, the photo-identification data available support the findings of population differentiation for snubfin dolphins in this study. Research at Cygnet Bay suggests a high degree of site fidelity for snubfin dolphins, with >80% of individuals resighted across ≥ three of a total of four×one-month field seasons from 2012–2013 (Brown *et al.*, unpublished data). Photo-identification records for snubfin dolphins in Roebuck Bay also suggest a high degree of site fidelity with >40 individuals resighted multiple times over a range of seasons between 2007 and 2012 (Thiele *et al*., unpublished data). Additionally, these data have not revealed any movement of snubfin dolphin individuals between Cygnet Bay and Roebuck Bay to date (Brown *et al*., unpublished data; Thiele *et al*., unpublished data).

Studies on snubfin and humpback dolphins from the east coast of Australia have revealed either a majority of individuals regularly using the same discrete area from year to year [24], or strong site fidelity within resident populations [26], [29]. These patterns of site fidelity support our finding of genetic structuring of snubfin and humpback dolphins of north-western Australia. We acknowledge that distances between sampling locations were large (>200 km) and, therefore, cannot rule out that a pattern of isolation-by-distance could explain the significant genetic structuring. However, we cannot test for isolation-by distance based on only two sampling locations for each species.

While acknowledging differences in distances between studies, our results support the conclusions of Cagnazzi [30] for humpback dolphins along the east coast of Queensland, where significant genetic differentiation was found between populations separated by *ca*. 200 km, but also between populations separated by only a few kilometres [29], [30].

In contrast to our current results and those of Cagnazzi [30], a study of humpback dolphins in Chinese waters found no evidence of population structure among three resident populations, each separated by approximately 500 km of coastline [65]. Potentially suitable habitat (river mouths) is distributed along much of the coastline [95], and a maximum dispersal distance of 300 km has been recorded for an individual in this region [96]. This suggests that a stepping-stone pattern of gene flow may be occurring, to a level sufficient to prevent differentiation. It was also suggested that gene flow might be of a recently interrupted form, where insufficient time has passed for detectable differentiation to develop [65].

Humpback dolphins have been observed in areas between the sampling locations of this current study [40], although their distribution along the north-western Australian coast remains poorly understood. Individual movements of up to 130 km have been recorded off the east coast of Australia [30]. No obvious natural geographic barriers to dispersal exist along the 350 km of coastline between the Dampier Archipelago and North West Cape, so the significant genetic differentiation found between animals at these two locations may be a result of their geographic separation exceeding individual dispersal distances.

The identification of genetic population structure in snubfin dolphins on the Queensland coast by Cagnazzi [30] was somewhat restricted by the distribution of sampling locations. No structure was found between three relatively close populations (within a 200 km stretch of coast), although significant differentiation was found at a much greater separation of approximately 600 km. Cygnet Bay and Roebuck Bay are separated by approximately 250 km of coastline. Based on our current understanding of the habitat requirements of snubfin dolphins [31], no obvious barriers to dispersal exist between the two sites: the coastline is currently undeveloped and shallow inshore waters are present throughout. Sightings between the two sites are largely restricted to anecdotal reports of small groups immediately north of Roebuck Bay [40]. Two months of boat survey effort along a 30 km stretch of coast between the two sites revealed a low encounter rate of just two sightings of the same pair of snubfin individuals (Brown *et al.*, unpublished data). The maximum reported distance travelled by an individual snubfin dolphin is 70 km [30], suggesting that the geographic distance between Cygnet Bay and Roebuck Bay is likely a key driver of the restricted gene flow documented here.

While barriers to dispersal are rarely obvious in marine habitats, significant genetic structure over relatively small spatial scales has been observed in numerous species of coastal dolphins (e.g. *Tursiops* spp. [97]–[100]; *Cephalorhynchus hectori* spp. [56]; *Sotalia guianesis* [101]). For bottlenose dolphins (*Tursiops* spp.), a range of environmental, habitat and resource specialisation and social factors have been suggested as drivers of fine-scale population structure (e.g. [99], [100], [102]–[105]).

### Effective population size and evidence of bottlenecks

For successful conservation strategies, it is important to have an understanding of the effective population size (*N_e_*), which provides an indicator of the number of individuals contributing genes to the next generation [106]. The effective population size is usually lower than the census size, and by definition describes the rate of inbreeding accumulation and loss of genetic diversity [87]. A rule of thumb suggests that *N_e_* should not fall below 50 in the short-term and should be above 500 in the long-term [107]. Mace & Lande [13] suggest that, subject to additional criteria (e.g. population decline), a population of *N_e_*<50 should be considered in a critical state (i.e. 50% probability of extinction within five years or two generations). We found that *N_e_* estimates are close to this theoretical lower limit for snubfin dolphins at Cygnet Bay (*N_e_* = 49.1, 95% CI 28.6–112.1) and Roebuck Bay (56.0, 95% CI 24.3–77180.6). While this may raise conservation concerns, the wide confidence intervals indicate considerable uncertainty in these estimates, particularly for Roebuck Bay. This suggests that sample sizes are too small to accurately estimate *N_e_* and limits our interpretation of these results.

The results on recent bottlenecks are ambiguous for the four sampling sites we investigated. Depending on the mutation model, we obtained significant and non-significant results for each site. The graphical allele frequency distortion method indicated a mode shift of humpback dolphins at the North West Cape. The presence of a recent bottleneck is supported by a low mtDNA diversity (one haplotype) identified at this sampling location. However, under the two-phased model of mutation there was no indication for a recent bottleneck at the North West Cape. The results of our assessments of recent bottlenecks and *N_e_* should be interpreted with caution due to ambiguity and large confidence intervals, respectively.

### Hybridisation

We found strong genetic evidence that the suspected hybrid found at Cygnet Bay is the offspring of a snubfin dolphin mother and a humpback dolphin father. While we found that alleles at three microsatellite loci (Table 3) were not shared between the hybrid and humpback dolphins, it is most likely that these alleles also exist in humpback dolphins, but have not been sampled as yet (because only five samples were collected from this species at Cygnet Bay). The absence of these alleles in our samples could also be due to the presence of null alleles, in particular, for the locus LobsDi9 (Table 3).

This is the first documented case of hybridisation between snubfin and humpback dolphins. The hybrid is a female, seemingly fully grown and in good body condition, which associates primarily with snubfin dolphins – her maternal species. Despite a predominance of male sterility among mammalian hybrids (e.g. [108]), there are several examples of fertility among female cetacean hybrids (e.g. within the Genus *Phocoena* [9]; *Balaenoptera* [109], [110]; and *Psuedorca×Tursiops* [34]) and one record of fertility of a male hybrid of the *Globicephala* genus [38]. In the absence of any evidence of the reproductive history of the snubfin-humpback hybrid identified here, no assessment of her fertility can be made at this stage.

Snubfin and humpback dolphins are sympatric across much of their range, occasionally form mixed groups, and aggressive-sexual inter-specific interactions have been documented [39]. Snubfin-humpback dolphin associations within Cygnet Bay appear to be uncommon and typically affiliative, although one observation of repeated mating attempts by a male humpback dolphin with a female snubfin has been recorded (Brown *et al.*, unpublished data). Frequent hybridisation has been documented between Dall’s (*Phocoena dalli*) and harbour (*Phocoena phocoena*) porpoises in a localised area of the northeast Pacific [9]. In all hybrids examined, Willis *et al*. [9] revealed Dall’s porpoise to be the maternal species, and suggested that the highly promiscuous male harbour porpoise’s indiscriminate pursuit of females of either species could be a driving factor of this hybridisation. In this region, the harbour porpoise is the rarer species, having apparently declined in recent decades [111]. Humpback dolphins, identified as the paternal species of the hybrid in the current study, are the least numerous of the three dolphin species within Cygnet Bay (Brown *et al*., unpublished data). We hypothesise that the observed propensity of humpback dolphins to initiate aggressive-sexual interactions with snubfin dolphins [39], along with a low availability of conspecific potential mates at the Cygnet Bay study site, are potential drivers of the hybrid dolphin reported here.

Our discovery of a snubfin-humpback dolphin hybrid shows that these two sympatric species are capable of inter-generic hybridisation. There are no indications that snubfin and humpback dolphins interbreed regularly from our data, and molecular studies of these animals on the east coast of Australia have not revealed any evidence of hybridisation, to date [30]. However, total sample sizes are small for both species, with limited survey effort throughout the majority of their range in Australia. This phenomenon likely represents a low-frequency, natural hybridisation, facilitated by a fragmented distribution and potentially low abundance [8], [9], [45]. Further isolation of already fragmented populations may facilitate further hybridisation and, hence, raise conservation concerns [43].

### Conservation and management implications

The definition of populations, stocks or management units (MUs) is typically based on ecological or evolutionary criteria, or a combination of the two [112]. Many different definitions of a population are in use and the criteria used vary according to the purpose for which a population is being defined [112]. Genetic data have been widely used to examine the structure of cetacean populations and to make recommendations on the identification of MUs (e.g. [98], [113], [114]). Indeed, the level of differentiation we have identified, in terms of significant F_ST_ values, supports the criteria for separate MUs as proposed by Moritz [115]. However, many authors argue that identifying MUs from genetic data alone is unwise (e.g. [112], [116], [117]), particularly via the use of F_ST_ alone to infer gene flow as it relies on several simplifying assumptions, which typically are not met for natural populations [118], [119]. Furthermore, an absence of historical gene flow may not correspond to current demographic isolation, yet it is the contemporary movement of animals which may be more pertinent in conservation and management actions [118]. While a combination of demographic, ecological and genetic data will provide the most robust assessments of MUs (e.g. [117], [120], [121]), such inter-disciplinary approaches require considerable resources and lengthy time-frames [120].

Palsbøll *et al*. [118] advocate an approach to defining MUs based on a predefined threshold level of genetic divergence, rather than the rejection of panmixia. They encourage a demographic interpretation, with the dispersal rate (i.e. migration rates) of individuals of greater relevance to conservation and management than historical gene flow. A commonly cited threshold for demographic dependence is at least 10% exchange [122]. Among our results, the estimated upper confidence intervals for migrant proportions were ≤0.1 for snubfin dolphins, which supports, with reasonable confidence, the notion of separate MUs based on dispersal rates. The large confidence intervals around our estimated migration rates for humpback dolphins include the value of 0.1, making it difficult to determine if the two sampled locations represent independent MUs based upon proposed dispersal thresholds [118]. A larger number of samples is required to more accurately estimate contemporary migration rates of humpback dolphins.

While based on limited sample sizes, our results suggest that north-western Australian snubfin and humpback dolphins may exist as metapopulations of small, genetically largely isolated population fragments. As such, they are vulnerable to genetic characteristics associated with small, fragmented populations; these include the accumulation of deleterious mutations, the loss of genetic diversity through random genetic drift, inbreeding depression, and a reduced ability to adapt to environmental change [12]. Our data, when combined with our (albeit limited) understanding of their movements, ecology and population structure from elsewhere in their range, suggest that the sampled populations are somewhat isolated and should be managed accordingly. For both species, further data are required to gain a better understanding of their genetic population structure, movements and demographics. However, it would seem appropriate to manage the two sampled populations of snubfin dolphins at Cygnet and Roebuck Bays as independent MUs. Despite the uncertainty around contemporary migration rates between humpback dolphins at the Dampier Archipelago and North West Cape, there is significant population structure and limited gene flow between these sampled populations; in light of the threat of coastal development in this region (described below), we recommend a precautionary approach of managing the sampled populations as independent MUs until further data become available.

Concerns have been raised with regard to the rate of industrial development along the coast of north-western Australia given the lack of appropriate baseline data on inshore dolphins in this region [25], [40], [41]. A resources boom, focussing on offshore hydrocarbon reserves and terrestrial mineral deposits, has been driving the rapid development of port and coastal processing facilities. The scale of these developments and, in particular, the volume of dredging, is large by global standards. Individual projects are responsible for tens of millions of cubic metres of seafloor dredging; combined dredging volumes for the region are in the hundreds of millions of cubic metres [25], [123]. Several such developments (either constructed, under-construction and in-planning) lie within 100 km of the Dampier Archipelago sampling site, while a plan for the world’s largest liquefied natural gas processing facility was approved (but subsequently abandoned by the proponents) at a site 50 km north of Roebuck Bay [124].

For tropical inshore dolphins, which are reliant upon the near-shore environment, the habitat modification associated with such coastal development presents multiple pathways for potential effects [14]. For snubfin and humpback dolphins, in particular, data deficiencies are precluding assessment of their conservation status and, therefore, their effective management in this rapidly developing region [25], [40]. Given the results presented here, we recommend that conservation actions should include efforts to reduce extinction risk by maintaining effective population size and gene flow. Further restrictions on gene flow or a reduction in effective population size may compromise their evolutionary potential and, therefore, the longevity, of these populations.

### Recommendations

We recommend the following conservation actions:

1. *Broad-scale baseline data collection*. Our results are based on a limited sample size, representing a small proportion of the several thousand kilometres of coastline of north-western Australia. The collection of baseline data on the distribution and abundance of inshore dolphins is required to identify and characterise local populations. Similarly, a greater number of biopsy samples across a broader geographic range are required to gain a more detailed understanding of their population genetic structure and connectivity.
2. *Better understanding and protection of identified local populations.* Each local population identified in this study is likely to serve a critical role as a stepping stone for gene flow among a fragmented metapopulation. For each local population, baseline data should be collected on abundance, effective population size, habitat use and potential or realised threatening processes. Data should inform management plans, which identify potential threats to the population, assess the vulnerability of the population against IUCN Red List Criteria, and make recommendations on actions required. Management plans should seek to minimise anthropogenic threats to local populations.
3. *Protection of movement corridors between local populations.* The occasional dispersal of breeding individuals between local populations results in the gene flow required to maintain the evolutionary potential of these small populations of dolphins. As such, proponents of development along the coast should consider their environmental footprint in relation to local populations of snubfin and humpback dolphins and the influence their activities (e.g. prolonged acoustic disturbance) may have on the movement of animals between populations, regardless of the density of animals observed in the vicinity. We strongly urge management agencies and decision-makers (e.g. the Government of Western Australia’s Environmental Protection Authority and the Department of Parks and Wildlife) to consider the potential cumulative impacts of multiple developments and other threatening processes.

## Supporting Information

Figure S1 **Δk plot for snubfin dolphins (A) and humpback dolphins (B).** In B, Δk peaks at k = 4 indicating that the most likely number of clusters equals 4.
(TIF)

Figure S2 **Structure plot including all samples used for this study.** OH = snubfin dolphin, *suspected hybrid, SC = humpback dolphin, TA = bottlenose dolphin, CY = Cygnet Bay, RB = Roebuck Bay, DA = Dampier Archipelago, NWC = North West Cape.
(TIF)

Figure S3 **Allele frequency distribution visualising potential mode-shift distortion.** The figures are based on 12 microsatellite loci for snubfin dolphins and 13 microsatellite loci for humpback dolphins.
(TIF)

Figure S4 **Neighbour-Joining tree of all haplotypes (based on 416 bp) identified in the three resident dolphin populations at Cygnet Bay.** TA = bottlenose dolphin, SC = humpback dolphin, OH = snubfin dolphin. The percentage of replicate trees in which the associated taxa clustered together in the bootstrap test (1000 replicates) is shown next to the branches.
(TIF)

Table S1 **Locus-specific microsatellite characteristics for snubfin dolphins.** N_A_ = Number of Alleles, N_E_ = Number of effective Alleles, N_P_ = Private Alleles, H_E_ = expected heterozygosity, H_O_ = observed heterozygosity, * = excluding monomorphic loci.
(DOCX)

Table S2 **Locus-specific microsatellite characteristics for humpback dolphins.** N_A_ = Number of Alleles, N_E_ = Number of effective Alleles, N_P_ = Private Alleles, H_E_ = expected heterozygosity, H_O_ = observed heterozygosity, * = excluding monomorphic loci.
(DOCX)

Table S3 ***P*** **values (from Wilcoxon sign-rank test) and presence of mode shifts indicating whether dolphins have recently undergone a bottleneck at our sampling locations.** Visualisations of potential mode shifts are shown in Figure S3. *H*: heterozygosity; IAM: infinite allele model; SMM: stepwise mutation model; *statistically significant result (*P*<0.05): ^¶^assessed by BOTTLENECK.
(DOCX)

Table S4 **Genotype data for snubfin, humpback and hybrid dolphins.** OH = snubfin dolphin, SC = humpback dolphin, CY = Cygnet Bay, DA = Dampier Archipelago, EX = North West Cape, RB = Roebuck Bay, H = hybrid dolphin.
(XLSX)
