# Correction: Population Differentiation and Hybridisation of Australian Snubfin (*Orcaella heinsohni*) and Indo-Pacific Humpback (*Sousa chinensis*) Dolphins in North-Western Australia

**DOI:** 10.1371/journal.pone.0109228

**Published:** 2014-09-19

**Authors:** The PLOS ONE Staff

There is an error in the legend for Figure 4. The complete, correct Figure 4 legend is:

**Figure 4:**
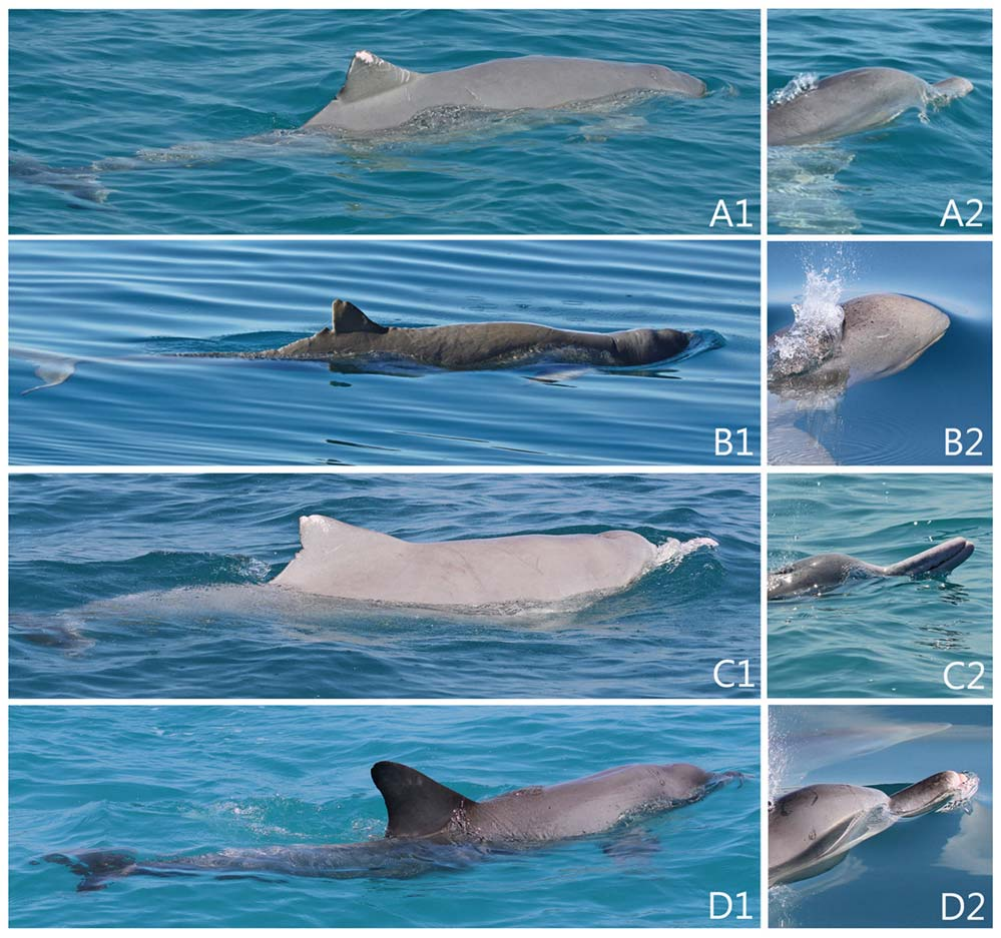
Images of hybrid (A1–2), adult snubfin (B1–2), humpback (C1–2) and bottlenose (D1–2) dolphins encountered at Cygnet Bay. Left images show relative dorsal proportions; right images compare head/rostrum characteristics.
