# Supplementary figures and images for: Population Differentiation and Hybridisation of Australian Snubfin (Orcaella heinsohni) and Indo-Pacific Humpback (Sousa chinensis) Dolphins in North-Western Australia
Source: PLoS One. 2014 Jul 2;9(7):e101427. doi: 10.1371/journal.pone.0101427 (PMC4079686; doi:10.1371/journal.pone.0101427)

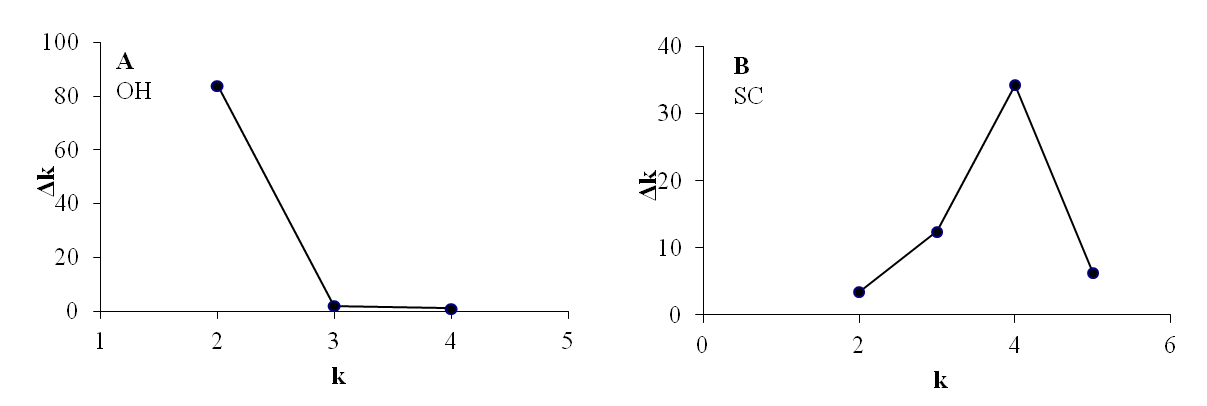

Supplement: Figure S1 — Δk plot for snubfin dolphins (A) and humpback dolphins (B). In B, Δk peaks at k = 4 indicating that the most likely number of clusters equals 4. (TIF) [file pone.0101427.s001.tif]

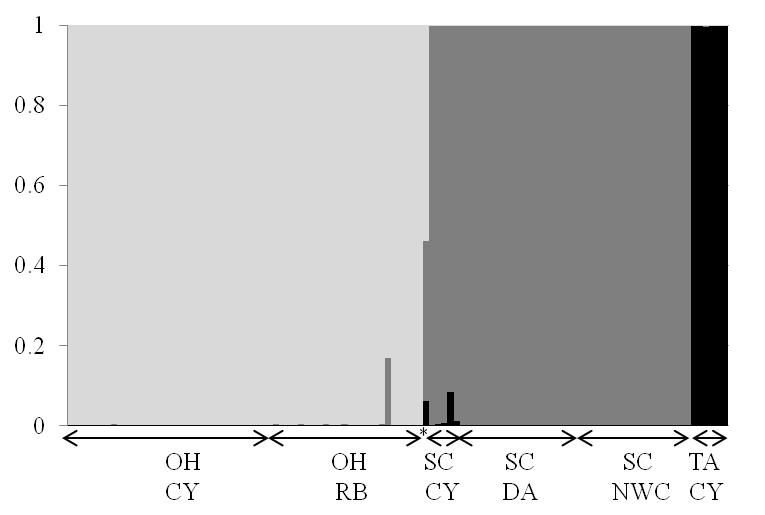

Supplement: Figure S2 — Structure plot including all samples used for this study. OH = snubfin dolphin, *suspected hybrid, SC = humpback dolphin, TA = bottlenose dolphin, CY = Cygnet Bay, RB = Roebuck Bay, DA = Dampier Archipelago, NWC = North West Cape. (TIF) [file pone.0101427.s002.tif]

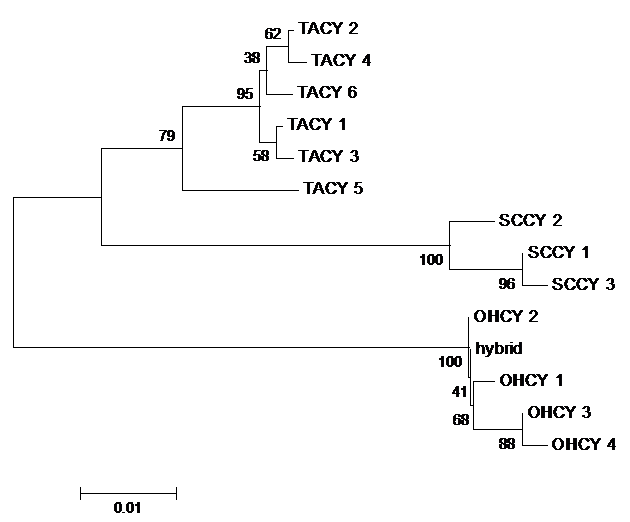

Supplement: Figure S4 — Neighbour-Joining tree of all haplotypes (based on 416 bp) identified in the three resident dolphin populations at Cygnet Bay. TA = bottlenose dolphin, SC = humpback dolphin, OH = snubfin dolphin. The percentage of replicate trees in which the associated taxa clustered together in the bootstrap test (1000 replicates) is shown next to the branches. (TIF) [file pone.0101427.s004.tif]
