# Supplementary material for: Population Differentiation and Hybridisation of Australian Snubfin (Orcaella heinsohni) and Indo-Pacific Humpback (Sousa chinensis) Dolphins in North-Western Australia
Source: PLoS One. 2014 Jul 2;9(7):e101427. doi: 10.1371/journal.pone.0101427 (PMC4079686; doi:10.1371/journal.pone.0101427)
Supplement: Table S1 — Locus-specific microsatellite characteristics for snubfin dolphins. NA = Number of Alleles, NE = Number of effective Alleles, NP = Private Alleles, HE = expected heterozygosity, HO = observed heterozygosity, * = excluding monomorphic loci. (DOCX) [file pone.0101427.s005.docx]

**Table S1.** Locus-specific microsatellite characteristics for snubfin dolphins.

|  | **Cygnet Bay** (*N* = 32) | | | | | **Roebuck Bay** (*N* = 25) | | | | |
| --- | --- | --- | --- | --- | --- | --- | --- | --- | --- | --- |
| **Locus** | **N_A_** | **N_E_** | **N_P_** | **H_E_** | **H_O_** | **N_A_** | **N_E_** | **N_P_** | **H_E_** | **H_O_** |
| **DIrFCB4** | 11 | 5.306 | 5 | 0.812 | 0.813 | 9 | 5.919 | 3 | 0.831 | 1 |
| **DIrFCB5** | 5 | 2.332 | 1 | 0.571 | 0.567 | 4 | 2.935 | - | 0.659 | 0.737 |
| **LobsDi7.1** | 4 | 3.012 | 1 | 0.668 | 0.625 | 4 | 2.290 | 1 | 0.563 | 0.522 |
| **LobsDi9** | 1 | 1 | - | 0 | 0 | 1 | 1 | - | 0 | 0 |
| **LobsDi19** | 2 | 1.789 | - | 0.441 | 0.406 | 2 | 1.814 | - | 0.449 | 0.440 |
| **LobsDi21** | 5 | 3.391 | - | 0.705 | 0.688 | 5 | 3.086 | - | 0.676 | 0.760 |
| **LobsDi24** | 3 | 2.073 | - | 0.518 | 0.500 | 3 | 1.580 | - | 0.367 | 0.280 |
| **LobsDi39** | 1 | 1 | - | 0 | 0 | 1 | 1 | - | 0 | 0 |
| **SCA9** | 4 | 3.489 | - | 0.713 | 0.875 | 4 | 2.890 | - | 0.654 | 0.727 |
| **SCA22** | 3 | 2.476 | - | 0.596 | 0.656 | 3 | 2.237 | - | 0.553 | 0.522 |
| **SCA27** | 7 | 2.790 | 1 | 0.642 | 0.719 | 10 | 6.187 | 3 | 0.838 | 0.739 |
| **SCA39** | 3 | 1.679 | - | 0.404 | 0.375 | 3 | 1.788 | - | 0.441 | 0.360 |
| **Tex5** | 2 | 1.519 | - | 0.342 | 0.250 | 2 | 1.891 | - | 0.471 | 0.520 |
| **Tex7** | 2 | 1.897 | - | 0.473 | 0.500 | 2 | 1.956 | - | 0.489 | 0.550 |
| **Mean*** | 4.25 | 2.65 |  | 0.57 | 0.58 | 4.25 | 2.88 |  | 0.58 | 0.60 |

N_A_ = Number of Alleles, N_E_ = Number of effective Alleles, N_P_ = Private Alleles, H_E_ = expected heterozygosity, H_O_ = observed heterozygosity, * = excluding monomorphic loci
