# Supplementary material for: Population Differentiation and Hybridisation of Australian Snubfin (Orcaella heinsohni) and Indo-Pacific Humpback (Sousa chinensis) Dolphins in North-Western Australia
Source: PLoS One. 2014 Jul 2;9(7):e101427. doi: 10.1371/journal.pone.0101427 (PMC4079686; doi:10.1371/journal.pone.0101427)
Supplement: Table S2 — Locus-specific microsatellite characteristics for humpback dolphins. NA = Number of Alleles, NE = Number of effective Alleles, NP = Private Alleles, HE = expected heterozygosity, HO = observed heterozygosity, * = excluding monomorphic loci. (DOCX) [file pone.0101427.s006.docx]

**Table S2.** Locus-specific microsatellite characteristics for humpback dolphins.

|  | **Cygnet Bay** (*N* = 5) | | | | | **Dampier** **Archipelago** (*N* = 19) | | | | | **North West Cape** (*N* = 18) | | | | |
| --- | --- | --- | --- | --- | --- | --- | --- | --- | --- | --- | --- | --- | --- | --- | --- |
| **Locus** | **N_A_** | **N_E_** | **N_P_** | **H_E_** | **H_O_** | **N_A_** | **N_E_** | **N_P_** | **H_E_** | **H_O_** | **N_A_** | **N_E_** | **N_P_** | **H_E_** | **H_O_** |
| **DIrFCB4** | 5 | 4.000 | 1 | 0.750 | 1 | 5 | 2.234 | 1 | 0.552 | 0.611 | 2 | 1.220 | - | 0.180 | 0.200 |
| **DIrFCB5** | 2 | 2.000 | - | 0.500 | 1 | 1 | 1 | - | - | - | 2 | 1.061 | - | 0.057 | 0.059 |
| **LobsDi7.1** | 4 | 2.941 | 1 | 0.660 | 0.600 | 5 | 3.238 | 1 | 0.691 | 0.526 | 3 | 1.684 | - | 0.406 | 0.375 |
| **LobsDi9** | 2 | 1.220 | 1 | 0.180 | 0.200 | 1 | 1 | - | - | - | 1 | 1.000 | - | 0.000 | 0.000 |
| **LobsDi19** | 4 | 3.846 | - | 0.740 | 1 | 6 | 2.843 | - | 0.648 | 0.737 | 6 | 3.461 | - | 0.711 | 0.706 |
| **LobsDi21** | 2 | 1.220 | 1 | 0.180 | 0.200 | 2 | 1.111 | - | 0.100 | 0.105 | 2 | 1.117 | - | 0.105 | 0.111 |
| **LobsDi24** | 5 | 4.167 | 1 | 0.760 | 1 | 3 | 1.875 | - | 0.467 | 0.632 | 5 | 3.880 | - | 0.742 | 0.667 |
| **LobsDi39** | 3 | 2.778 | 1 | 0.640 | 1 | 2 | 1.385 | - | 0.278 | 0.333 | 2 | 1.301 | - | 0.231 | 0.267 |
| **SCA9** | 6 | 3.333 | 1 | 0.700 | 0.800 | 5 | 3.153 | 1 | 0.683 | 0.579 | 8 | 4.208 | 2 | 0.762 | 0.556 |
| **SCA22** | 5 | 3.571 | 1 | 0.720 | 0.800 | 5 | 2.725 | 1 | 0.633 | 0.526 | 5 | 3.400 | - | 0.706 | 0.647 |
| **SCA27** | 2 | 1.471 | - | 0.320 | 0.400 | 3 | 1.174 | - | 0.148 | 0.158 | 3 | 1.325 | - | 0.245 | 0.167 |
| **SCA39** | 4 | 2.381 | 1 | 0.580 | 0.600 | 3 | 2.105 | - | 0.525 | 0.684 | 3 | 2.219 | - | 0.549 | 0.444 |
| **Tex5** | 1 | 1 | - | - | - | 1 | 1 | - |  | - | 1 | 1.000 | - | 0.000 | 0.000 |
| **Tex7** | 2 | 1.471 | 1 | 0.320 | 0.400 | 2 | 1.166 | - | 0.142 | 0.154 | 2 | 1.057 | - | 0.054 | 0.056 |
| Mean* | 3.54 | 2.65 |  | 0.54 | 0.69 | 3.73 | 2.09 |  | 0.44 | 0.46 | 3.58 | 2.16 |  | 0.40 | 0.35 |

N_A_ = Number of Alleles, N_E_ = Number of effective Alleles, N_P_ = Private Alleles, H_E_ = expected heterozygosity, H_O_ = observed heterozygosity, * = excluding monomorphic loci
