# Supplementary material for: Population Differentiation and Hybridisation of Australian Snubfin (Orcaella heinsohni) and Indo-Pacific Humpback (Sousa chinensis) Dolphins in North-Western Australia
Source: PLoS One. 2014 Jul 2;9(7):e101427. doi: 10.1371/journal.pone.0101427 (PMC4079686; doi:10.1371/journal.pone.0101427)
Supplement: Table S3 — P values (from Wilcoxon sign-rank test) and presence of mode shifts indicating whether dolphins have recently undergone a bottleneck at our sampling locations. Visualisations of potential mode shifts are shown in Figure S3. H: heterozygosity; IAM: infinite allele model; SMM: stepwise mutation model; *statistically significant result (P<0.05): ¶assessed by BOTTLENECK. (DOCX) [file pone.0101427.s007.docx]

**Table S3.** *P* values (from Wilcoxon sign-rank test) and presence of mode shifts indicating whether dolphins have recently undergone a bottleneck at our sampling locations. Visualisations of potential mode shifts are shown in Figure S3.

|  | **Two-tailed *P* values for *H* excess or deficiency** | | | **Mode shift^¶^** |
| --- | --- | --- | --- | --- |
|  | **IAM** | **SMM** | **TPM** |  |
| **Snubfin dolphins** |  |  |  |  |
| Cygnet Bay (n = 32) | <0.01* | 0.79 | 0.04* | no |
| Roebuck Bay (n = 25) | <0.01* | 0.20 | <0.01* | no |
| **Humpback dolphins** |  |  |  |  |
| Dampier Archipelago  (n = 19) | 0.76 | <0.01* | 0.28 | no |
| North West Cape (n = 18) | 1.0 | 0.03* | 0.23 | yes |

*H*: heterozygosity; IAM: infinite allele model; SMM: stepwise mutation model; *statistically significant result (*P* < 0.05): **^¶^**assessed by BOTTLENECK
